# Phase II trial of docetaxel in advanced or metastatic endometrial cancer: a Japanese Cooperative Study

**DOI:** 10.1038/sj.bjc.6602817

**Published:** 2005-10-18

**Authors:** N Katsumata, K Noda, S Nozawa, R Kitagawa, R Nishimura, S Yamaguchi, D Aoki, N Susumu, H Kuramoto, T Jobo, K Ueki, M Ueki, I Kohno, K Fujiwara, Y Sohda, F Eguchi

**Affiliations:** 1 Department of Medical Oncology, National Cancer Center Hospital, 104-0045 Tokyo, Japan; 2 Kinki University, Osakasayama, Japan; 3 Department of Obstetrics and Gynecology, School of Medicine, Keio University,160-8582 Tokyo, Japan; 4 Department of Gynecology, Hyogo Medical Center for Adults, 673-8558 Akashi, Japan; 5 Department of Obstetrics and Gynecology, Kitasato University, 228-8555 Sagamihara, Japan; 6 Department of Obstetrics and Gynecology, Osaka Medical College, 569-8686 Takatsuki, Japan; 7 Department of Obstetrics and Gynecology,Kawasaki Medical School, 701-0192 Kurashiki, Japan; 8 Department of Obstetrics and Gynecology, Aso Iizuka Hospital, 820-8505 Iizuka, Japan

**Keywords:** docetaxel, endometrial cancer, phase II

## Abstract

The purpose of this study was to determine whether docetaxel has antitumour activity in patients with advanced or recurrent endometrial carcinoma. Chemotherapy-naïve or previously treated patients (one regimen) with histopathologically documented endometrial carcinoma and Eastern Cooperative Oncology Group performance status ⩽2 entered the study. Docetaxel 70 mg m^−2^ was administered intravenously on day 1 of a 3-week cycle up to a maximum of six cycles. If patients responded well to docetaxel, additional cycles were administered until progressive disease or unacceptable toxicity occurred. Of 33 patients with a median age of 59 years (range, 39–74 years) who entered the study, 14 patients (42%) had received one prior chemotherapy regimen. In all, 32 patients were evaluable for efficacy, yielding an overall response rate of 31% (95% confidence interval, 16.1–50.0%); complete response and partial response (PR) were 3 and 28%, respectively. Of 13 pretreated patients, three (23%) had a PR. The median duration of response was 1.8 months. The median time to progression was 3.9 months. The predominant toxicity was grade 3–4 neutropenia, occurring in 94% of the patients, although febrile neutropenia arose in 9% of the patients. Oedema was mild and infrequent. Docetaxel has antitumour activity in patients with advanced or recurrent endometrial carcinoma, including those previously treated with chemotherapy; however, the effect was transient and accompanied by pronounced neutropenia in most patients.

Most patients with endometrial cancer are diagnosed at an early stage when surgery alone may result in cure. However, the outcome for women with advanced stage or recurrent disease is poor and rarely curable. Both single-agent and combination regimens of chemotherapy have been studied in women with advanced endometrial carcinoma. Currently, no standard chemotherapy regimen for endometrial cancer exists, but single-agent doxorubicin is active, with responses observed in up to one-third of previously untreated patients (Moore *et al*, 1991). Other single agents with modest activity include cisplatin (Thigpen *et al*, 1984a, 1989) and carboplatin (van Wijk *et al*, 2003). Although the response rates with the combination doxorubicin−cisplatin appear to be higher than those achieved with either agent alone, there is no evidence that survival is any longer with combination therapy. In the Gynecologic Oncology Group (GOG) trial comparing doxorubicin alone with doxorubicin−cisplatin, the response rates and progression-free survival were better with the combination regimen (42 *vs* 25%, 5.7 *vs* 3.8 months, respectively), but overall survival (OS) had not significantly improved (Thigpen *et al*, 2004).

The taxanes, paclitaxel and docetaxel, are potent chemotherapeutic agents that block tubulin depolymerisation, leading to the inhibition of microtubule dynamics, and have significant clinical efficacy for various solid tumours. Paclitaxel has been evaluated as an active agent for endometrial cancer (Ball *et al*, 1996; Lissoni *et al*, 1996; Lincoln *et al*, 2003). However, preclinical data show that docetaxel has increased potency and an improved therapeutic index compared with paclitaxel (Bissery *et al*, 1995), and its short 1-h infusion time offers a substantial clinical advantage over the prolonged infusion durations required with paclitaxel. Docetaxel and paclitaxel also have substantially different toxicity profiles. In particular, docetaxel has a significant lower incidence of neurotoxicity in comparison to paclitaxel (Hsu *et al*, 2004).

The present phase II trial was designed to evaluate the clinical efficacy and tolerability of docetaxel 70 mg m^−2^ in patients with advanced or recurrent endometrial cancer.

## PATIENTS AND METHODS

### Eligibility criteria

Eligible patients aged between 20 and 74 years, with a life expectancy in excess of 3 months, and Eastern Cooperative Oncology Group (ECOG) Performance Status (PS) of 0−2 had histologically documented primary stage III, IV or recurrent endometrial carcinoma. Tumours were staged according to the International Federation of Gynecology and Obstetrics criteria. All patients had measurable disease according to the response evaluation criteria in solid tumours (RECIST) (Therasse *et al*, 2000). Measurable lesions defined unidimensionally were ⩾20 mm using conventional imaging, or ⩾10 mm with spiral computed tomographic scan. Patients were either chemotherapy-naive or had received one prior chemotherapy regimen for endometrial cancer, with 4 weeks between prior therapy and study treatment. Prior treatment with a taxane was not allowed. Adequate organ function was required for study entry: neutrophil count ⩾2000 *μ*l^−1^, platelet count ⩾100 000 *μ*l^−1^, haemoglobin ⩾9.0 g dl^−1^, serum bilirubin level ⩽1.5 mg dl^−1^, normal hepatic function (asparate aminotransferase (AST), alanine aminotransferase (ALT) and alkaline phosphatase (ALP) levels ⩽2.5 times upper limit of the institutional normal (ULN)), serum creatinine level ⩽1.5 mg dl^−1^, PaO_2_ ⩾60 mmHg and normal electrocardiogram. Patients with any of the following conditions were excluded from the study: sarcoma component, active infection, severe heart disease, interstitial pneumonitis, past history of hypersensitivity, peripheral neuropathy, malignant or benign effusions requiring drainage, active brain metastasis, or active concomitant malignancy. All patients gave informed consent before entering this study, which was approved by the institutional review boards at all participating institutions.

### Treatment schedule

Docetaxel 70 mg m^−2^ was infused over a 1–2-h period. The treatment was repeated every 3 weeks unless there was documented disease progression or unacceptable toxicity. Prophylactic medications for nausea, vomiting or hypersensitivity reactions were given if these symptoms occurred. No routine premedication was given for hypersensitivity reactions and fluid retention during the first cycle of treatment. The patient's physician identified all hypersensitivity reactions and, if deemed necessary, the investigator administered premedication drugs.

Treatment was delayed for up to 3 weeks in the event of toxicity, but was restarted when the neutrophil count was ⩾1500 *μ*l^−1^, platelet count ⩾100 000 *μ*l^−1^, AST/ALT/ALP levels ⩽2.5 times ULN, and neuropathy or oedema ⩽grade 1. Docetaxel dosage was reduced by 10 mg m^−2^ if febrile neutropenia occurred, if there was bleeding with grade 3−4 thrombocytopenia requiring a platelet transfusion, or if a patient experienced any grade 3−4 nonhaematologic toxicities except nausea, vomiting, anorexia, fatigue, alopecia or hypersensitivity.

### Response and toxicity evaluation

The tumour response was assessed according to the standard RECIST criteria (Therasse *et al*, 2000). Target lesions included all measurable lesions up to a maximum of five lesions per organ and 10 lesions in total. Complete response (CR) was defined as the complete disappearance of all target and nontarget lesions, with no development of new disease. Partial response (PR) was defined as a reduction by ⩾30% in the sum of the longest diameter of target lesions. Complete response or PRss were confirmed by repeat assessments performed no less than 4 weeks after the criteria for response were first met. Progressive disease (PD) was defined as an increase by ⩾20% in the sum of the longest diameter of all target lesions, or the appearance of one or more new lesions and/or unequivocal progression of existing, nontarget lesions. Stable disease (SD) was defined as neither sufficient lesion shrinkage to qualify for a PR, nor sufficient increase to qualify for PD. Best response was defined as the most CR achieved by a patient (thus, each patient had a single best response: CR, PR, SD or PD), and the date of best response was the date it was first detected. Time to progression (TTP) was defined as the time from the first medication to the date of a PD event or death (due to endometrial cancer or study drugs). All tumours were radiographically assessed for response every 6 weeks. An independent response review committee (IRRC) evaluated all tumour responses after the investigators had completed their judgement.

Toxicities were evaluated with respect to incidence and severity using National Cancer Institute common toxicity criteria (NCI-CTC, version 2.0) (Trotti *et al*, 2000).

### Statistical consideration

Assuming a response rate of 20%, the study was designed with 80% power such that the lower limit of the 95% confidence interval (CI) for the estimate of the response rate was greater than 0.05. A sample size of 32 evaluable patients was required.

The primary end point was overall tumour response (determined by the IRRC) with the corresponding 95% CI using the exact binominal method for the evaluable population. The secondary end point of this study was safety. The Kaplan–Meier (KM) method was used to determine the TTP and median survival time (MST) in the evaluable population.

## RESULTS

### Patient characteristics

A total of 33 patients were enrolled on the study from April 2001 to October 2003 and one patient was unevaluable as a result of having received prior treatment with paclitaxel and doxorubicin−platinum regimens. The median age of the intent to treat (ITT) population (*n*=33) was 59 years (range 39–74) and 70% patients had ECOG PS 0 (Table 1). Several patients had unfavourable histologic characteristics: adenosquamous features (three) and uterine papillary serous cancers (two). Most patients (88%) had undergone total abdominal hysterectomy and bilateral salpingo-oophorectomy, and one-third of patients had prior radiotherapy. Of those patients who had received prior chemotherapy (*n*=14), 10 had received combination doxorubicin−platinum in combination, three had received platinum alone and one had received oral fluorouracil. All 33 patients were evaluated for toxicity and survival, while 32 patients were evaluated for response and TTP.

### Treatment delivery

Overall, 32 patients received a total of 133 cycles of docetaxel and the median number of cycles of docetaxel was four (range, 1−13). Five patients (15%) experienced dose reductions for the following reasons: two patients experienced febrile neutropenia (in one patient this occurred twice) and three patients had grade 3 nonhaematologic toxicities: diarrhoea (occurred twice in one patient), hyperglycaemia, hyperkalaemia and supraventricular tachycardia.

### Response

**Figure 1:**
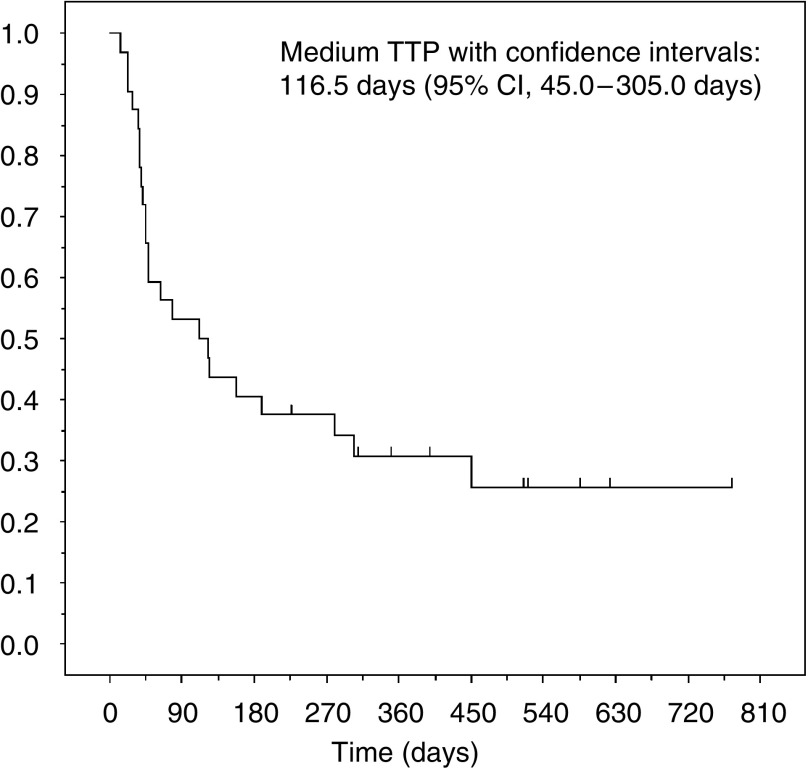
KM curve of estimated TTP.

Table 2 presents the assessment of response to treatment. Two patients, one who was chemotherapy-naïve and the other who had received prior therapy, were not assessable for response because they had received only one cycle of treatment. Before evaluation by the IRRC, primary physicians had reported two CRs and nine PRs. The IRRC judged one CR as a PR, two PR as SD and one SD as a PR. Therefore, the overall response rate for 10 of 32 patients was 31% (95% CI, 16.1–50.0%). Of 13 patients who had prior chemotherapy, three (23%) achieved a PR: two had received doxorubicin−platinum and one platinum alone. The histologic analysis revealed responses among the following tumour types: endometrioid adenocarcinoma (6 of 25 patients), squamous differentiated adenocarcinoma (1 of 3), papillary serous (2 of 2) and undifferentiated cancer (1 of 1). The median time for the onset of effect was 2.0 months (range, 0.7–4.5) and the median duration of response was 1.8 months (range, 0.9−4.6). The median follow-up time was 17.6 months (range, 1.7−36.3) and median TTP was 3.9 months (95% CI, 1.5−10.2 months) (Figure 1). Median survival time was 17.8 months (95% CI, 7.4−22.0 months).

### Safety and toxicity

In all, 33 patients were assessable for toxicity (Table 3). Also, 31 (94%) patients experienced grade 3 or 4 neutropenia, and three (9%) developed febrile neutropenia. Nonhaematologic toxicities included grade 3 anorexia and vomiting experienced by some patients (18 and 9%, respectively). One patient experienced grade 3 peripheral neuropathy (sensory and motor) after five treatment cycles. Three patients terminated the study as a consequence of the following toxicities: infection with *Mycobacterium avium* complex (one), grade 4 hypersensitivity reaction despite premedication with dexamethasone (one) and grade 3 oedema with pleural effusion after six treatment cycles (one). All three patients recovered after receiving recommended medical treatment. There were no treatment-related deaths.

## DISCUSSION

At initial diagnosis, only a small percentage of endometrial cancer patients have recurrent or advanced disease with distant metastases, and therefore a multicentre trial is essential for the accrual of patients. This multicentre phase II trial, although relatively small in sample size, clearly demonstrated that docetaxel is active in the treatment of endometrial cancer. Toxicity was manageable and predominantly haematologic.

Taxanes have shown activity in this setting previously, with paclitaxel demonstrating overall response rates of 27–37% when used as a single agent in endometrial cancer (Ball *et al*, 1996; Lissoni *et al*, 1996; Lincoln *et al*, 2003). Combination chemotherapy with paclitaxel and carboplatin or cisplatin has resulted in response rates of 50–56% (Dimopoulos *et al*, 2000; Hoskins *et al*, 2001; Scudder *et al*, 2005). However, a GOG randomised trial of women with advanced or recurrent endometrial carcinoma, in which the combination paclitaxel−doxorubicin was compared with doxorubicin−cisplatin, showed that the paclitaxel arm did not result in an improved outcome (Fleming *et al*, 2000). A subsequent GOG study, in which the combination paclitaxel, doxorubicin and cisplatin (TAP) with G-CSF was compared with doxorubicin−cisplatin, showed that the TAP arm yielded a better response (57 *vs* 34%; *P*<0.01), progression-free survival (median, 8.3 *vs* 5.3 months; *P*<0.01) and OS (median, 15.3 *vs* 12.3 months; *P*=0.037) than the control arm. However, more grade 3 neuropathy (12 *vs* 1%) and congestive heart failure were observed with TAP than with doxorubicin−cisplatin (Fleming *et al*, 2004). In light of this imbalance between efficacy and toxicity, TAP has not been accepted as the standard chemotherapy regimen in routine clinical practice.

Docetaxel has a toxicity profile that is different from paclitaxel. In particular, neurotoxicity occurs at a low incidence with docetaxel. In our study, only one patient developed grade 3 neuropathy-sensory and recovered in several weeks. While fluid retention is a distinctive toxicity of docetaxel, this can be prevented using premedication (Piccart *et al*, 1997); in our trial, one patient developed pleural effusion since the routine premedication with corticosteroids was not applied.

Several studies have reported on second-line chemotherapy for endometrial cancer. Two phase II trials of second-line paclitaxel report response rates of 27% (12 out of 44) and 37% (7 out of 19) (Lissoni *et al*, 1996; Lincoln *et al*, 2003). An older report describes a 30% response rate to second-line high-dose cisplatin (3 mg kg^−1^) among 13 patients (Deppe *et al*, 1980). With the exception of these studies, response rates to second-line chemotherapy are uniformly less than 20% and most are less than 10% (Slayton *et al*, 1982, 1988; Stehman *et al*, 1983; Thigpen *et al*, 1984b, 1986; Homesley *et al*, 1986; Asbury *et al*, 1990; Muss *et al*, 1991, 1993; Sutton *et al*, 1994; Rose *et al*, 1996; Muggia *et al*, 2002). In our study, 23% of pretreated patients (3 out of 13) had a PR to docetaxel, suggesting that it too is active as second-line therapy.

In conclusion, this multicentre phase II trial shows that docetaxel is active in the treatment of chemotherapy-naïve and chemotherapy pretreated patients with advanced or recurrent endometrial cancer and possesses a manageable toxicity profile; however, the effect was transient and accompanied by pronounced neutropenia in most patients. The exploration of the efficacy of docetaxel combinations in phase III studies for the treatment of endometrial cancer is of great interest and will be initiated.

## Figures and Tables

**Table 1:** Patient characteristics

**Characteristic**	**No. of patients (*n*=33)**
*Age, years*	
Median	59
Range	39–74
	
*ECOG performance status*	
0	23
1	9
2	1
	
*Disease status*	
Stage III, IV	9
Recurrent	24
	
*Histology*	
Endometrioid	26
Adenocarcinoma with squamous differentiated	3
Papillary serous	2
Adenocarcinoma, unspecified	1
Undifferentiated	1
	
*Tumour grade*	
1	11
2	11
3	6
Unknown	5
	
*Prior treatment*	
Surgery	29
Radiotherapy	9
Hormonal therapy	5
	
*Prior chemotherapy*	
None	19
Doxorubicin and platinum	9
Platinum alone	3
Others	2

ECOG=Eastern Cooperative Oncology Group.

**Table 2:** Best response (RECIST criteria) to docetaxel

	**Prior chemotherapy (*n*=13)**		**No prior chemotherapy (*n*=19)**		**Total (*n*=32)**	
**Response**	**No. of patients**	**%**	**No. of patients**	**%**	**No. of patients**	**%**
Complete response	0	0	1	5	1	3
Partial response	3	23	6	32	9	28
Stable disease	4	31	5	26	9	28
Progressive disease	5	38	6	32	11	34
Not assessable	1	8	1	5	2	6
						
ORR (95% CI)		23 (5.0–53.8)		37 (16.3–61.6)		31 (16.1–50.0)

ORR=overall response rate; CI=confidence interval.

**Table 3:** Adverse effects

	**NCI-CTC grade (*n*=33)**									
	**1**		**2**		**3**		**4**		**3−4**	
**Toxicities**	**No.**	**%**	**No.**	**%**	**No.**	**%**	**No.**	**%**	**No.**	**%**
Neutrophils	1	3	0	0	10	30	21	64	31	94
Haemoglobin	11	33	11	33	1	3	1	3	2	6
Lymphopenia	1	3	14	42	11	33	—	11	33	
Platelets	6	18	1	3	0	0	0	0	0	0
Alopecia	5	15	26	79	—	—	—			
Fatigue	13	39	7	21	3	9	0	0	3	9
Anorexia	12	36	5	15	6	18	0	0	6	18
Nausea	16	49	6	18	2	6	—	2	6	
Vomiting	7	21	3	9	3	9	0	0	3	9
Diarrhoea	14	42	3	9	3	9	0	0	3	9
Constipation	2	6	10	30	4	12	0	0	4	12
Stomatitis	3	9	5	15	1	3	0	0	1	3
Febrile neutropenia	—	—	3	9	0	0	3	9		
Infection	0	0	3	9	0	0	0	0	0	0
Oedema	7	21	3	9	1	3	0	0	1	3
Neuropathy-motor	1	3	0	0	1	3	0	0	1	3
Neuropathy-sensory	9	27	2	6	1	3	0	0	1	3
Supraventricular arrhythmia	0	0	0	0	1	3	0	0	1	3
Allergic reaction	3	9	0	0	0	0	1	3	1	3
Rash/desquamation	6	18	5	15	1	3	0	0	1	3
Injection site reaction	5	15	2	6	0	0	0	0	0	0
Nail changes	4	12	0	0	—	—	—			
AST	9	27	3	9	0	0	0	0	0	0
ALT	8	24	2	6	0	0	0	0	0	0
Hypokalaemia	0	0	—	3	9	0	0	3	9	

NCI-CTC=National Cancer Institute common toxicity criteria; AST=asparate aminotransferase; ALT=alanine aminotransferase.

Present NCI-CTC grade 3−4 in >5% patients and breakdown if possible by whether patient had prior chemotherapy.

## Appendix

The following institutions (with principal investigators) participated in this study: Sapporo Medical University, Sapporo, Satoru Sagae; Niigata University, Niigata, Kenichi Tanaka; Tochigi National Hospital, Utsunomiya, Masaaki Kikuchi; National hospital Organization Saitama Hospital, Wako, Mikio Mikami; National Cancer Center Hospital, Tokyo, Noriyuki Katsumata; Tokyo Women's Medical University, Tokyo, Hiroaki Ohta; School of medicine, Keio University, Tokyo, Daisuke Aoki; St. Marianna University School of Medicine, Kawasaki, Kazushige Kiguchi; Kitasato University, Sagamihara, Hiroyuki Kuramoto; Nagoya City University, Nagoya, Atsushi Arakawa; Fujita Health University, Toyoake, Yasuhiro Udagawa; Aichi Cancer Center Hospital, Nagoya, Kazuo Kuzuya; Osaka Medical College, Takatsuki, Ken Ueki; Kinki University, Osakasayama, Hiroshi Hoshiai; Hyogo Medical Center for Adults, Akashi, Ryuichiro Nishimura; Wakayama Medical University, Wakayama, Katsuji Kokawa; Okayama University, Okayama, Junichi Kodama; Okayama Red Cross General Hospital, Okayama, Kohei Ejiri; Kawasaki Medical School, Kurashiki, Ichiro Kohno; National Kyushu Cancer Center, Fukuoka, Toshiaki Saito; Kyusyu University, Fukuoka, Toshio Hirakawa; Fukuoka University, Fukuoka, Toru Hachisuga; University of Occupational and Environmental Health, Kitakyuushu, Naoyuki Toki; Kurume University, Kurume, Toshiharu Kamura; Kurume University Medical Center, Kurume, Naofumi Okura; Aso Iizuka Hospital, Iizuka, Yasuhito Sohda; Saga University, Saga, Tsuyoshi Iwasaka; Kagoshima City Hospital, Kagoshima, Masayuki Hatae; Tokyo Teishin Hospital, Tokyo, Hiroki Hata.
